# Small Intestinal Obstruction Caused by Anisakiasis

**DOI:** 10.1155/2013/401937

**Published:** 2013-12-26

**Authors:** Yuichi Takano, Kuniyo Gomi, Toshiyuki Endo, Reika Suzuki, Masashi Hayashi, Toru Nakanishi, Ayumi Tateno, Eiichi Yamamura, Kunio Asonuma, Satoshi Ino, Yuichiro Kuroki, Masatsugu Nagahama, Kazuaki Inoue, Hiroshi Takahashi

**Affiliations:** Division of Gastroenterology, Showa University Fujigaoka Hospital, 1-30 Fujigaoka, Aoba-ku, Yokohama-shi, Kanagawa 227-0043, Japan

## Abstract

Small intestinal anisakiasis is a rare disease that is very difficult to diagnose, and its initial diagnosis is often surgical. However, it is typically a benign disease that resolves with conservative treatment, and unnecessary surgery can be avoided if it is appropriately diagnosed. This case report is an example of small intestinal obstruction caused by anisakiasis that resolved with conservative treatment. A 63-year-old man admitted to our department with acute abdominal pain. A history of raw fish (sushi) ingestion was recorded. Abdominal CT demonstrated small intestinal dilatation with wall thickening and contrast enhancement. Ascitic fluid was found on the liver surface and in the Douglas pouch. His IgE (RIST) was elevated, and he tested positive for the anti-*Anisakis* antibodies IgG and IgA. Small intestinal obstruction by anisakiasis was highly suspected and conservative treatment was performed, ileus tube, fasting, and fluid replacement. Symptoms quickly resolved, and he was discharged on the seventh day of admission. Small intestinal anisakiasis is a relatively uncommon disease, the diagnosis of which may be difficult. Because it is a self-limiting disease that usually resolves in 1-2 weeks, a conservative approach is advisable to avoid unnecessary surgery.

## 1. Case Report

A 63-year-old previously healthy male was brought to our emergency department because of sudden, severe abdominal pain and vomiting. The patient had eaten raw fish (sushi) two days before admission. His blood pressure was 130/66 mmHg, pulse was 103 BPM, and body temperature was 36.1°C. He had severe epigastric tenderness; however, no rebound tenderness or guarding was observed. A blood test revealed that his white blood cell count was 9800/*μ*L and CRP was 5.0 mg/dL, indicating inflammation. His eosinophil count was within the normal range at 3.0%. His IgG was also within the normal range (870–1,700) at 871 mg/dL; however, his IgE (RIST) was elevated at 253 IU/mL (normal value ≤ 170). He tested positive for the anti-*Anisakis* antibodies IgG and IgA.

**Figure 1:**
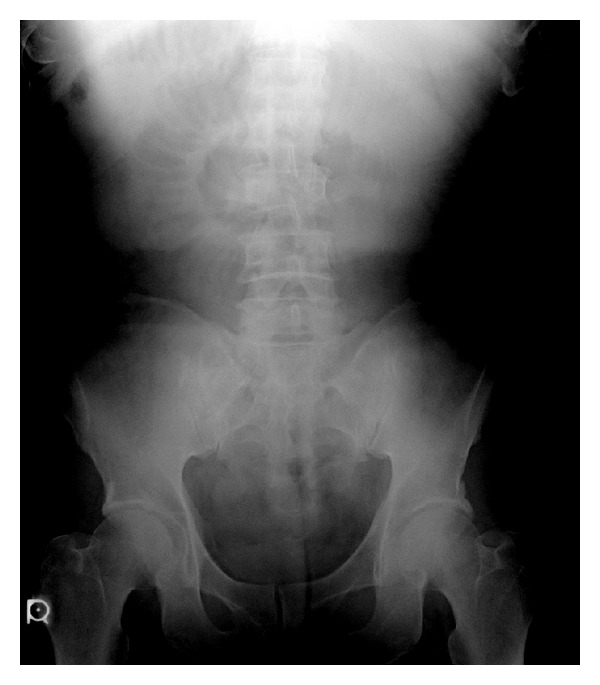
A plain abdominal X-ray (in decubitus) revealed a prominent sentinel loop.

**Figure 2:**
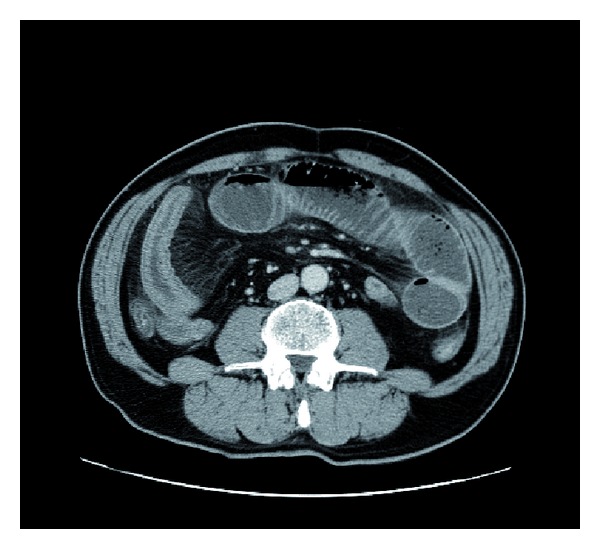
Contrast-enhanced abdominal CT findings demonstrated small intestinal dilatation with wall thickening and contrast enhancement.

**Figure 3:**
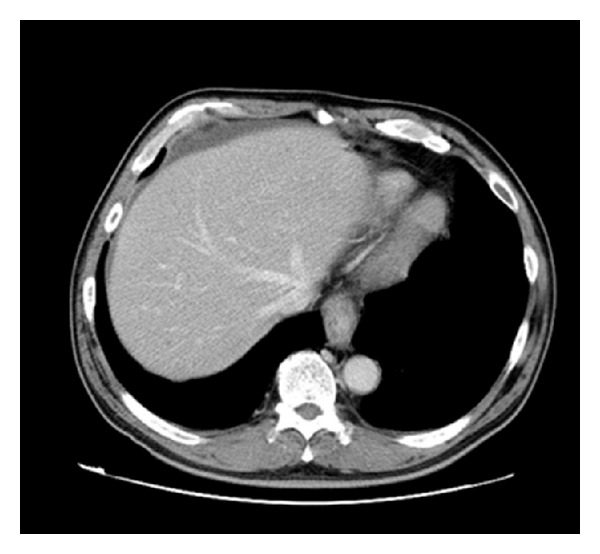
Ascitic fluid was found on the liver surface and in the Douglas pouch.

A plain abdominal X-ray (in decubitus) revealed a prominent sentinel loop (Figure 1). Contrast-enhanced abdominal CT findings were small intestinal dilatation with wall thickening and contrast enhancement (Figure 2). Ascitic fluid was found on the liver surface and in in the Douglas pouch (Figure 3).

**Figure 4:**
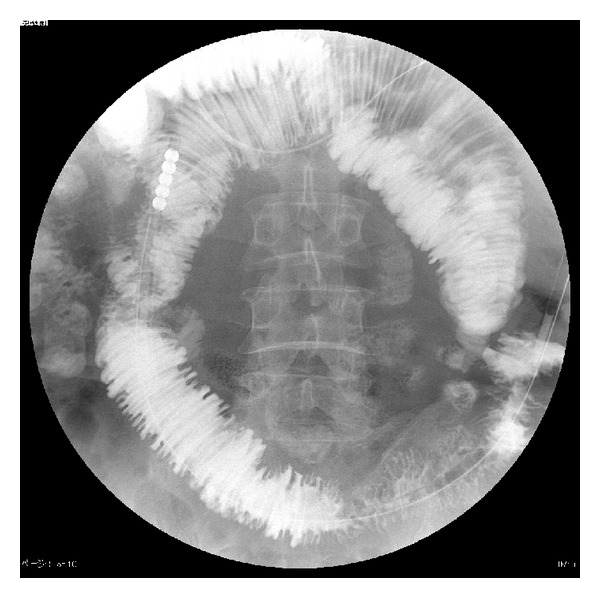
A contrast-enhanced image of the ileus tube shows the normal small intestine.

We diagnosed the case as small intestinal obstruction by anisakiasis and decided to prioritize conservative treatment. On the first day of admission, we promptly inserted an ileus tube and treated the disease by fasting and fluid replacement. Symptoms quickly resolved, and on the fifth day of admission, we took a contrast-enhanced image of the ileus tube (Figure 4). There were no obvious abnormalities of the small intestine; therefore, we removed the ileus tube. Because the patient was doing well after that, he was discharged on the seventh day of admission.

We conducted study on human participants with the approval of the University Ethics Committee.

## 2. Discussion

Anisakiasis, first reported by Van Thiel in 1960 [1], is a fish-borne parasitic disease caused by consumption of raw or undercooked fish or cephalopods contaminated by third-stage larvae of the Anisakidae family, in particular *Anisakis simplex*, *A. pegreffii*, and *Pseudoterranova decipiens*. Every year, approximately 20,000 anisakiasis cases are reported worldwide. More than 90% of these cases are from Japan; most others occur in Spain, The Netherlands, and Germany, depending on the habits of raw fish consumption [2].

Anisakiasis commonly involves the stomach, and rarely the intestine. A large study conducted in Japan [3] revealed that only 567 (4.5%) of 12,586 anisakiasis cases involved the intestine. Therefore, the diagnosis of intestinal anisakiasis can be difficult. Recording the history of recent raw fish consumption is vital for correct diagnosis. In cases with this history presenting with acute abdominal pain, the possibility of anisakiasis should be considered in the differential diagnosis [4, 5].

Abdominal ultrasonography (AUS) and computed tomography scanning (CT) are useful in the diagnosis of intestinal anisakiasis [6–8]. Typical CT findings include a relatively long segment of the symmetric wall thickening with luminal narrowing, diffuse contrast enhancement of the involved segment, and ascites [6, 7]. Ido et al. [8], in a study conducted in 12 patients, confirmed three common findings on AUS in patients with anisakiasis: (1) presence of a relatively large amount of ascitic fluid; (2) dilatation of the small intestine; and (3) marked localized edema of Kerckring's folds. Unfortunately, in our case, AUS was not performed.

Compared with gastric anisakiasis, more patients with small-intestinal anisakiasis exhibited leukocytosis (76.7%), eosinophilia (43.3%), and elevated C-reactive protein levels (3.4 ± 3.2 mg/dL) [9]. These findings may also be helpful.

An elevated titer of anti-*Anisakis* antibodies in blood is sensitive (100%) but not very specific (50%) marker because of cross reactions with proteins in other parasites (*Ascaris*,* Toxocara*, and *Echinococcus*), microorganisms, insects, and plants [8, 10, 11]. The measurement of the anti-*Anisakis* antibody titer may take some time, and this mode of detection may be ineffective during the early phase [8, 12]; nevertheless it is useful tool for the diagnosis.

In a study describing 15 small intestinal anisakiasis cases, Castán reported a decrease in the need for surgery over time. In the period from 1989 to 1996, surgery was required in 6/6 (100%) cases, while from 1997 to 2001, this proportion decreased to 2/9 (22%) [13]. Ishida et al. [14] suggested that conservative treatment is preferable over surgery if intestinal anisakiasis is correctly diagnosed. In their report on 3 small intestinal anisakiasis cases, surgery was required in the first case because preoperative diagnosis was difficult. However, in the other 2 cases, correct diagnosis of small intestinal anisakiasis was obtained by reference to their experience with the first case. Symptoms improved with conservative treatment, fasting, and transfusion. Another recent study [15] demonstrated that surgery is required in only 7% (14/201) of intestinal anisakiasis cases, which is usually a self-limiting disease curable with conservative treatment. Surgery for small intestinal anisakiasis must be avoided except in cases with complications of perforation, volvulus, or severe long-segment small intestinal stenosis [16, 17].

## 3. Conclusion

Small intestinal anisakiasis is a rare disease, the diagnosis of which may be difficult. Because it is a self-limiting disease that usually resolves in 1-2 weeks, a conservative approach is advisable to avoid unnecessary surgery.
